# El laboratorio en el diagnóstico multidisciplinar del desarrollo sexual anómalo o diferente (DSD): III) Marcadores bioquímicos y genéticos en los 46,XY IV) Propuestas para el diagnóstico diferencial de los DSD

**DOI:** 10.1515/almed-2020-0120

**Published:** 2021-05-24

**Authors:** Maria Luisa Granada, Laura Audí

**Affiliations:** Department of Clinical Biochemistry, Hospital Germans Trias i Pujol, Autonomous University of Barcelona, Badalona, España; Growth and Development Research Group, Vall d’Hebron Research Institute (VHIR), Center for Biomedical Research on Rare Diseases (CIBERER), Instituto de Salud Carlos III, Barcelona, Catalonia, España

**Keywords:** desarrollo sexual anómalo o diferente (DSD), diagnóstico bioquímico, diagnóstico genético, DSD 46,XY, algoritmo diagnóstico

## Abstract

**Objetivos:**

El desarrollo sexual anómalo o diferente (DSD) con cariotipo 46,XY incluye anomalías en el desarrollo gonadal y/o genital (externo y/o interno).

**Contenido:**

Los marcadores bioquímicos útiles para el diagnóstico diferencial de los DSD con cariotipo 46,XY incluyen las hormonas del eje hipotálamo-hipófiso gonadal como son las gonadotropinas LH y FSH (en condiciones basales o tras la estimulación con LHRH), la hormona anti-Mülleriana, la inhibina B, el factor insulinoide tipo 3 y las hormonas esteroideas de origen suprarrenal (se incluirá la hormona hipofisaria ACTH) y testicular (cortisol, aldosterona y sus precursores, testosterona y sus precursores, dihidrotestosterona y estradiol). Las hormonas esteroideas se analizarán en condiciones basales o tras la estimulación con ACTH (hormonas adrenales) y/o con HCG (hormonas testiculares). Los patrones de variación de las distintas hormonas dependerán de la causa y la edad de cada paciente. El diagnóstico molecular debe incluir el análisis de un gen candidato, un panel de genes o el análisis de un exoma completo.

**Perspectivas:**

El diagnóstico diferencial de los DSD con cariotipos 46,XX ó 46,XY debe ser multidisciplinar, incluyendo los antecedentes clínicos, morfológicos, de imagen, bioquímicos y genéticos. Se han elaborado numerosos algoritmos diagnósticos.

## III. Marcadores bioquímicos y genéticos en los DSD 46,XY

Cuando el cariotipo es 46,XY y existe alguna discordancia a nivel del desarrollo gonadal y/o genital, es necesario evaluar hormonas relacionadas con el desarrollo testicular y con la síntesis de andrógenos y la hormona anti-Mülleriana (AMH) [1]. El eje hipotálamo-hipófiso-testicular (HHT) es muy activo durante la vida fetal. La hormona folículo-estimulante (FSH) controla la proliferación de células de Sertoli, responsables del aumento del volumen testicular y de la secreción de AMH y de la Inhibina B (INHB), mientras que la hormona luteinizante (LH) regula las células de Leydig responsables de la secreción de andrógenos, principalmente de la testosterona (T), así como de la proteína 3 similar a la insulina (INSL3), involucrada en el descenso de los testículos a las bolsas escrotales [2]. En el varón normal las concentraciones de T y de la LH y FSH en sangre al nacer son bajas aunque son superiores a las del sexo femenino, se elevan al final de la primera semana de vida llegando a un pico máximo alrededor de los 3 meses, a partir de los cuales descienden a niveles bajos alrededor de los 6 meses de vida y se mantienen así hasta el inicio de la pubertad [3]. Esta activación fisiológica del eje HHT se denomina “minipubertad” y es un período muy útil para el estudio de los pacientes con desarrollo sexual anómalo o diferente (DSD) en los primeros meses de vida. A partir del final del primer semestre de vida, tanto las gonadotropinas como la T presentan concentraciones séricas muy bajas y para valorar el eje HHT o la capacidad de síntesis de T por las células de Leydig deberá recurrirse a pruebas de estimulación de la hipófisis con el factor hipotalámico LHRH (GnRH) o sus análogos, o del testículo mediante un test de estimulación con gonadotropina coriónica (HCG). Sin embargo, disponemos de dos hormonas proteicas que son buenos marcadores de la función del túbulo seminífero durante la infancia y la prepubertad, la AMH y la INHB, ambas producidas por las células de Sertoli [4].

La AMH se secreta únicamente por las células de Sertoli y es un buen marcador de la disfunción testicular primaria [5]. Las concentraciones de AMH en el recién nacido (RN) y el niño prepuberal son muy elevadas y disminuyen al inicio de la pubertad, de forma inversa al aumento de T. Es necesario interpretar las concentraciones de AMH en función de la edad y del estadío puberal [6]. La AMH puede estar disminuída o ser indetectable en pacientes con disgenesia gonadal, pero está preservada en pacientes con afectación del compartimento intersticial (déficit aislado de la síntesis o de la acción de andrógenos).

La INHB se secreta por las células de Sertoli, pero no de manera exclusiva. Es el principal responsable de la inhibición de la FSH hipofisaria. Aunque no tiene una función específica en la diferenciación sexual fetal, es un marcador útil de función testicular. Las concentraciones de INHB son bajas en el RN y aumentan durante el primer mes de vida llegando a un pico máximo alrededor del 2º año. A partir de este momento, las concentraciones de INHB descienden, manteniéndose a concentraciones bajas pero detectables hasta el inicio de la pubertad [7], [8].

### 1) Desarrollo gonadal anómalo

En los 46,XY un desarrollo gonadal anómalo puede consistir en una disgenesia gonadal parcial (DGP) o completa (DGC), pero también puede existir un desarrollo ovotesticular o incluso ovárico (Tabla 1).

**Tabla 1:** Clasificación del desarrollo sexual anómalo o diferente (DSD) según los cromosomas sexuales XY presentes en el cariotipo.

DSD con cariotipo 46,XY	
1. Desarrollo gonadal anómalo	Disgenesia gonadal parcial (DGP) o completa (DGC) DSD ovotesticular DSD ovárico
2. Desarrollo genital anómalo por anomalías en la síntesis o en la acción de los andrógenos	**Anomalías en la síntesis de andrógenos:** Insensibilidad a la LH (aplasia/hipoplasia de células de Leydig) Déficit de 7-dehidrocolesterol reductasa (síndrome de Smith-Lemli-Opitz) Déficit de proteína StAR (hiperplasia suprarrenal congénita lipoidea) Déficit de colesterol desmolasa Déficit de 3β-hidroxi-esteroide deshidrogenasa tipo 2 Déficit de 17α-hidroxilasa/17–20 desmolasa Déficit de P450 oxidoreductasa Déficit de citocromo B5 Déficit en la esteroidogénesis de la vía trasera Déficit de 17β-hidroxi-esteroide deshidrogenasa tipo 3 Déficit de 5α-reductasa tipo 2 Hipospadias y/o criptorquidia aislados
	**Anomalías en la acción de los andrógenos:** Insensibilidad completa o parcial a los andrógenos Agentes terapéuticos o contaminantes medioambientales
3. Desarrollo genital anómalo por anomalías en la síntesis o en la acción de la hormona anti-Mülleriana (AMH)	**Persistencia de los conductos de Müller:** Déficit de hormona anti-Mülleriana Resistencia a la hormona anti-Mülleriana
4. Síndromes malformativos complejos	Síndromes malformativos con desarrollo genital anómalo (anomalías cloacales, síndrome de Aarskog, síndrome de Robinow, etc.) Crecimiento intrauterino retardado, precoz y severo

La DGC se presenta con genitales internos y externos femeninos. En lugar de gónadas existen unas cintillas bilaterales que no secretan andrógenos ni AMH. A nivel bioquímico se observa un hipogonadismo hipergonadotrópico con aumento de LH y de FSH, déficit de T y concentraciones indetectables de AMH y de INHB.

En las formas parciales (DGP), el grado de virilización depende de la cantidad de tejido gonadal funcional presente. La persistencia de restos derivados de los conductos de Müller refleja la producción defectuosa de AMH como un signo de disfunción de las células de Sertoli.

Gracias a las técnicas de secuenciación masiva cada vez se conocen más genes involucrados en la DGC y la DGP. En la Tabla 2 están descritos por orden alfabético. El primero descrito fue el gen *SRY.* Muchos de estos genes asocian la afectación de otros tejidos o sistemas (Tabla 2): *ARX, ATRX, CDKN1C, DHH, DMRT1, DMRT2, EMX2, FANCAFGFR2, GATA4, HHAT, LHX9, MYRF, NR5A1, PBX1, PPP2R3C, SAMD9, SOX9, TSPYL1, WT1.* En otros no se han descrito asociaciones (Tabla 2): las duplicaciones de *DAX1,* las mutaciones con efecto dominante en *DHX37,* en *MAP3K1,* en *WWOX* o en *ZNRF3,* las recesivas en *EFCAB6,* las monoalélicas o bialélicas en *ESR2* o en *SOX8,* las ligadas al cromosoma X en *FTHL17,* en *MAMLD1* o en *STARD8.*

**Tabla 2:** Diagnósticos clínicos y genes involucrados en el desarrollo sexual anómalo o diferente (DSD) de causa monogénica.

DSD con cariotipo 46,XY		
Diagnóstico clínico	Gen (locus)	OMIM (herencia) (fenotipo adicional)
1. DSD 46,XY por anomalías en el desarrollo gonadal: disgenesia gonadal completa (DGC) o parcial (DGP), DSD ovotesticular, DSD ovárico		
DGP	*ARX* (Xp21.3)	300215 (XL:D) (Lisencefalia, epilepsia, déficit intelectual)
DGP	*ATRX* (Xq21.1)	300032 (D:del) (déficit intelectual, α-talasemia)
DGC/DGP/DSD ovárico	*CBX2* (17q25.3)	602770 (AR) 613080 (AR) *CBX2.1*/(AD) *CBX2.2*
DGP/DGC	*CDKN1C* (11p15.4)	600856 (AD)/614732 (Síndrome IMAGe (retraso intrauterino del crecimiento, displasia metafisaria, hipoplasia suprarrenal congénita, anomalías genitales)
DGP/DGC	*DAX1 (NR0B1)* (Xp.21)	300018 (XL:dup)
DGP/DGC	*DHH* (12q13.12)	233420/607080 (AR/AD) (neuropatía minifascicular)
DGP/DGC	*DHX37* (12q24.31)	617362 (AD) (incluye síndrome de regresión testicular)
DGP/DGC	*DMRT1* (9p24.3) *DMRT2* (9p24.3)	602424 (AD:del) (con o sin déficit intelectual) 604935 (AD:del) (con o sin déficit intelectual)
DGC	*EFCAB6* (22q13.2-q13.31)	64800 (AR)
DGP	*EMX2* (10q26.11)	600035 (AD:del) (déficit intelectual, agenesia renal)
DGP/DGC	*ESR2* (14q23.2-q23.3)	601663 (bialélico y monoalélico)
DGP	*FANCA* (16q24.3)	606139 (AR) (anemia de Fanconi y microcefalia)
DGP/DGC	*FGFR2* (10q26.13)	176943 (AD) (craniosinostosis) (un solo caso descrito)
DGC	*FTHL17* (Xp21.2)	300308 (XL) (árbol familiar)
DGP/DGC	*GATA4* (8p23.1)	615542/600576 (AD) (con o sin cardiopatía congénita)
DGC	*HHAT* (1q32.2)	605743 (AR) (Un solo caso familiar descrito: talla baja, condrodisplasia generalizada, hipertrofia muscular, miopía, discreto déficit intelectual)
DGC	*LHX9* (1q31.3)	606066 (AD) (asocia malformaciones extremidades inferiores)
DGP	*MAMLD1 (CXOrf6)* (Xq28)	300120 (XL) (Hipospadias ligado al X)
DGP/DGC	*MAP3K1 (MEKK1)* (5q11.2)	613762/600692 (AD)
DG	*MYRF* (11q12.2)	608329 (AD)/618280 (AD) (Síndrome cardíaco urogenital)
DGP/DGC	*NR5A1* (9q33.3)	612965/184757 (AD)/(AR) (insuficiencia suprarrenal primaria/hipogonadismo hipogonadotropo muy infrecuente)
DGP	*PBX1* (1q23.3)	176310 (AD) (posible asociación con hipoplasia pulmonar, hipotonía, retraso psicomotor, sordera ± anomalía en las orejas, anomalías nefrourológicas, retraso de crecimiento)
DGC	*PPP2R3C* (14q13.2)	618419 (AR)/618420 (AD) (facies dismórfica, distrofia retiniana y miopatía; fallo espermatogénesis)
DG	*SAMD9* (7q21.2)	610456 (AD)/617053 Síndrome MIRAGE (mielodisplasia, infecciones, retraso de crecimiento, hipoplasia suprarrenal, fenotipo genital, enteropatía)
DGP/DGC	*SOX8* (16p13.3)	605923
DGP/DGC	*SOX9* (17q24.3)	114290 (AD) (displasia campomélica)
DGP/DGC	*SRY* (Yp11.2)	400044/480000 (D)
DGP/DGC	*STARD8* (Xq13.1)	300689 (XL)
DGP/DGC	*TSPYL1* (6q22.1)	604714/608800/(AR) (síndrome SIDDT “sudden infant death with dysgenesis of the testes”; muerte súbita infantil)
DGC/DSD ovotesticular/DSD ovárico	*WNT4* (1p36.12)	603490 (AD:dup)
DGP	*WT1* (11p.13)	607102 (AD) 1) 194072 (del 11.p13) (síndrome WAGR: tumor alto de de Wilms, retraso psicomotor y aniridia) 2) 194080 (inactivación) (síndrome de Denys-Drash: nefropatía, tumor alto de Wilms, riesgo de gonadoblastoma) 3) 136680 (corte y empalme) (síndrome de Frasier: nefropatía, riesgo alto de gonadoblastoma)
DGP	*WWOX* (16q23.1-q23.2)	605131 (AD:del) (un solo caso descrito con herencia materna)
DGP/DGC/DSD ovotesticular	*ZFPM2 (FOG2)* (8q23.1)	616067/603693 (AD) (con o sin cardiopatía congénita)
DGP/DGC	*ZNRF3* (22q12.1)	612062 (AD)

2. DSD 46,XY con anomalías en la biosíntesis de andrógenos o en su mecanismo de acción, hipospadias aislado, criptorquidia aislada		

Insensibilidad a LH/Gonadotrofina coriónica	*LHCGR* (2p16.3)	238320 (AR) (aplasia/hipoplasia de células de Leydig)
Déficit de 7-dehidro-colesterol reductasa	*DHCR7* (11q13.4)	270400 (AR) (síndrome de Smith-Lemli-Opitz)
Déficit de STAR (HSC lipoidea) 1) Forma clásica 2) Forma no-clásica	*STAR* (8p11.23)	201710 (AR) 1) Déficit suprarrenal y gonadal 2) Déficit suprarrenal
HSC por deficit de colesterol desmolasa	*CYP11A1* (15q24.1)	613743 (AR) (Déficit suprarrenal y gonadal)
HSC por deficit de 3β-hidroxi-esteroide deshidrogenasa tipo 2	*HSD3B2* (1p12)	201810 (AR) (Déficit suprarrenal y gonadal)
Déficit de 17α-hidroxilasa/17,20 desmolasa 1) HSC por déficit combinado 2) Déficit aislado de 17,20 desmolasa	*CYP17A1* (10q24.32)	202110 (AR) 1) HSC + Hipertensión + déficit gonadal 2) Déficit gonadal
Déficit de P450-oxidoreductasa	*POR* (7q11.23)	201750 (AR) (déficit de 17α-hidroxilasa, 21-hidroxilasa y aromatasa variables); (síndrome de Antley-Bixler syndrome, ± craniosinostosis)
Déficit de citocromo b5	*CYB5A* (18q22.3)	250790 (AR) (metahemoglobinemia tipo IV)
Déficit de la vía trasera de la esteroidogénesis	*AKR1C2* (10p15.1) *AKR1C4* (10p15.1)	614279 (AR) DSD por déficit de DHT con aparente déficit de 17,20-desmolasa y genes *CYP17A1* y *SRD5A2* normales
Déficit de 17β-hidroxi-esteroide-deshidrogenasa tipo 3 (17-ceto-reductasa)	*HSD17B3* (9q22.32)	264300 (AR) (déficit gonadal)
Déficit de 5α-reductasa tipo 2	*SRD5A2* (2p23.1)	264600 (AR)
Hipospadias ligado al X	*MAMLD1 (CXOrf6)* (Xq28)	300758 (XL) (hipospadias)
Hipospadias aislado	*ATF3* (1q32.3)	603148 (AD ??)
Criptorquidia	*INSL3* (19p13.11)	219050 (AD)
Criptorquidia	*RXFP2 (LGR8/GREAT/GPR106)* (13q13.1)	606655 (AD ??)
Insensibilidad a andrógenos: completa o parcial	*AR* (Xq12)	300068/312300/300633 (XL)

3. DSD 46,XY DSD por secreción o acción anómala de la hormona anti-Mülleriana (AMH)		

Déficit de AMH	*AMH* (19p13.3)	261550 (AR) (Persistencia de los conductos de Müller tipo I: hernia uterina inguinal y criptorquidia bilateral)
Insensibilidad a la AMH	*AMHR2* (12q13.13)	261550 (AR) (Persistencia de los conductos de Mülleria tipo II: hernia uterina inguinal y criptorquidia bilateral)

DSD, desarrollo sexual diferente; DG, disgenesia gonadal; DGP, disgenesia gonadal parcial; DGC, disgenesia gonadal completa; HSC, hiperplasia suprarrenal congénita; D, dominante; AD, autosómico dominante; AR, autosómico recesivo; XL, ligado al X; T, translocación; Dup, duplicación; Del, deleción; ¿?, desconocido; CNV, variación en número de copias.

El desarrollo ovotesticular u ovárico es muy poco frecuente y el diagnóstico es anátomo-patológico. Son marcadores genéticos conocidos las duplicaciones del gen *WNT4* (3 copias del gen) y las mutaciones monoalélicas en los genes *CBX2* o en *ZFPM2 (FOG2*) (Tabla 2). Para los tres se han descrito pacientes con DGC o con DGP.

### 2) Desarrollo genital anómalo por anomalías en la síntesis o acción de los andrógenos

En los 46,XY con gónadas testiculares la virilización de genitales internos y externos puede ser deficiente o nula por alteraciones en la síntesis o en la acción de los andrógenos (Tabla 1).

#### a) Anomalías en la síntesis de andrógenos

La deficiencia aislada de síntesis de andrógenos junto con un desarrollo testicular normal comprende la deficiente síntesis de T por las células de Leydig (resistencia a la LH, fallos en la biosíntesis de T (Tabla 1 [2a–j]) y el déficit de DHT por déficit de 5α-reductasa tipo 2 (Tabla 1 [2k]). En todos los casos, hay ausencia de útero porque los testículos secretan AMH normalmente. Algunas deficiencias en la biosíntesis de T testicular pueden afectar también la biosíntesis adrenal [9].

##### a-1) Insensibilidad a la LH (aplasia/hipoplasia de células de Leydig). Alteración del receptor de la LH

La disminución o falta de expresión del receptor para las gonadotropinas LH y HCG en las células del intersticio testicular, precursoras de las células de Leydig, provoca una hipoplasia o incluso la agenesia completa de células de Leydig. No se estimula la biosíntesis de T o está disminuida. Los genitales externos pueden ser completamente femeninos o ambiguos con hipospadias y criptorquidia. El perfil bioquímico incluye elevación basal de LH y aumento de respuesta a GnRH o sus análogos, junto con niveles de FSH normales y no aumentados. Las concentraciones de T son bajas y no responden al estímulo con HCG, siendo todos los precursores de T normales. Las concentraciones de AMH e INHB son normales, al ser normal la función de las células de Sertoli y su respuesta a la FSH [10], [11].

El diagnóstico molecular debe incluir la presencia de mutaciones bialélicas en el gen *LHCGR* (Tabla 2).

##### a-2) Déficit de 7-dehidrocolesterol reductasa (síndrome de Smith-Lemli-Opitz)

En el síndrome de Smith-Lemli-Opitz (SLO), el déficit del enzima 7-deshidrocolesterol reductasa (DHCR7) provoca un grave defecto en la biosíntesis del colesterol que conduce a concentraciones muy bajas de colesterol en plasma y gran aumento del precursor 7-deshidrocolesterol. Como el colesterol es indispensable en múltiples procesos del organismo, los niños con este déficit presentan un amplio espectro de malformaciones congénitas y discapacidad intelectual de expresión variable que incluye defectos de crecimiento, retraso mental con microcefalia, rasgos faciales característicos, sindactília del 2º y 3^er^ dedo del pie y alteraciones cardíacas, entre otras. Además, el colesterol es el precursor de todas las hormonas esteroideas por lo cual presentan insuficiencia suprarrenal, déficit de andrógenos y ambigüedad genital con micropene, hipospadias, criptorquidia y escroto bífido, que suele acompañarse de otras anomalías del tracto urinario y renales [12].

El diagnóstico molecular comporta el hallazgo de mutaciones bialélicas en el gen *DHCR7* (Tabla 2).

##### a-3) Déficit de proteína StAR (hiperplasia suprarrenal congénita lipoidea)

La proteína StAR (*Steroidogenic Acute Regulatory protein*) regula la transferencia del colesterol dentro de las mitocondrias constituyendo el paso limitante de la esteroidogénesis adrenal y gonadal. Los pacientes con déficit de StAR presentan hiperplasia suprarrenal congénita (HSC) grave, con déficit de glucocorticoides y mineralocorticoides que se manifiesta por hipoglicemia, hiperpigmentación de piel y mucosas, deshidratación y shock. A nivel bioquímico presentan déficit de cortisol y de aldosterona, así como de todos sus precursores, aumento de corticotropina (ACTH) y de la actividad de la renina plasmática (ARP), hiponatremia e hiperkaliemia. En el niño 46,XY el déficit de andrógenos testiculares se asocia a ambigüedad genital de diferentes grados con micropene, hipospadias, y criptorquidia. La T y sus precursores están disminuídos y no responden a la estimulación con HCG, y la LH está aumentada. La falta de metabolismo del colesterol provoca su acúmulo en forma de gotas lipídicas en la suprarrenal (HSC lipoidea) y en el intersticio del testículo [13].

Las formas clásicas presentan deficiencia suprarrenal y gonadal mientras que las formas menos graves o “no clásicas” sólo presentan deficiencia adrenal, pudiendo desarrollar una deficiencia gonadal pasada la pubertad [14]. En todos los casos existen mutaciones bialélicas en el gen *StAR* (Tabla 2).

##### a-4) Déficit de colesterol desmolasa

El enzima mitocondrial colesterol 20-22-desmolasa o P450scc (P450 *side chain cleavage*) cataliza la conversión del colesterol en pregnenolona, siendo un primer paso limitante de la esteroidogénesis adrenal y gonadal. Las consecuencias clínicas y bioquímicas de su deficiencia son similares a las del déficit de la proteína StAR, del que se diferenciará mediante el estudio genético que demuestre mutaciones bialélicas en el gen *CYP11A1* (Tabla 2). En este caso la afectación es siempre suprarrenal y gonadal.

##### a-5) Déficit de 3β-hidroxi-esteroide deshidrogenasa tipo 2 (HSD3B2)

La enzima HSD3B2 cataliza el paso de esteroides delta 5 (Δ5) (pregnenolona,17OH-pregnenolona y dehidroepiandrosterona [DHEA]) a esteroides delta 4 (Δ4). Las formas graves tienen una presentación clínica similar al déficit de proteína StAR o al de colesterol desmolasa. En las formas menos graves puede no haber déficit de mineralocorticoides ni clínica de pérdida salina. Se asocia a ambigüedad genital por déficit de T testicular. El diagnóstico específico se hace al demostrar un aumento de la 17OH-pregnenolona y de la DHEA basal o tras estimulación con ACTH; también está aumentado el sulfato de DHEA (DHEA-S). Los esteroides Δ4 (17-hidroxiprogesterona [17OH-P] y androstendiona) que deberían estar disminuídos, pueden estar elevados por la acción periférica de la isoenzima 3β-hidroxi-esteroide deshidrogenasa tipo I (HSD3B1), lo cual puede dificultar el diagnóstico diferencial con el déficit de 21-hidroxilasa y de 11β -hidroxilasa. Por la elevada frecuencia del déficit de 21-hidroxilasa, el diagnóstico diferencial suele incorporar el análisis de los genes *CYP21A2* y *HSD3B2*, aunque en los pacientes 46,XY la ambigüedad genital orienta mejor hacia el déficit de HSD3B2. Las mutaciones en *HSD3B2* son bialélicas (Tabla 2).

##### a-6) Déficit de 17α-hidroxilasa/17–20 liasa o desmolasa

Este enzima microsomal se expresa en el córtex adrenal y en las gónadas. La actividad de 17α-hidroxilación cataliza el paso de pregnenolona a 17-hidroxipregnenolona (17OH-Preg) y de progesterona (P) a 17OH-P. Su déficit impide la síntesis de cortisol, de andrógenos y de estrógenos. La actividad 17-20 liasa o desmolasa cataliza el paso de 17OH-Preg a DHEA y de 17OH-P a androstendiona, aunque este último paso es mucho menos eficiente [15], [16].

En el déficit combinado de 17α-hidroxilasa y 17–20 liasa, el aumento de ACTH debido al déficit de cortisol produce un gran incremento de pregnenolona, de P y de 11-desoxicorticosterona, la cual tiene acción mineralocorticoide, por lo que el cuadro clínico de HSC se acompaña de hipertensión arterial, alcalosis, hipokaliemia y supresión de la ARP. Los niños 46,XY presentan una falta de virilización de genitales externos e internos con hipoplasia de las estructuras derivadas de los conductos de Wolff y presencia de gónadas (testículos) intraabdominales. El déficit combinado es consecuencia de mutaciones bialélicas con efecto inactivador grave en el gen *CYP17A1* (Tabla 2). El déficit aislado de 17-20-liasa sólo provoca afectación de la esteroidogénesis gonadal y, por tanto, un DSD por déficit de T, con androstendiona y T disminuidas, pero P y 17OH-P normales o poco elevadas. Esta última forma aislada es consecuencia de mutaciones menos severas y con inactivación parcial del enzima (Tabla 2).

##### a-7) Déficit de P450-oxidorreductasa (POR)

El déficit de P450-oxidoreductasa (POR) también puede ser causa de HSC y de ambigüedad genital por déficit en la síntesis gonadal de T. Puede existir un déficit combinado de las actividades enzimáticas que utilizan este dador de electrones (CYP17, CYP21 y CYP19). La afectación de estas diversas actividades enzimáticas es variable y depende de las mutaciones que presente el gen *POR* [17]*.* El déficit de 21-hidroxilasa (CYP21) se manifiesta por un aumento de las concentraciones de 17OH-P y de su respuesta a la estimulación con ACTH, pero la disminución de CYP17 enmascara el déficit de CYP21 y provoca un déficit de T y androstendiona basales que puede evidenciarse durante la minipubertad o durante la prepubertad tras la estimulación con HCG. El diagnóstico molecular debe demostrar la presencia de mutaciones bialélicas en el gen *POR* (Tabla 2).

##### a-8) Déficit de citocromo B5

El citocromo B5 (cyb5) es una pequeña hemoproteína que tiene un papel modulador de la enzima P450 17α-hidroxilasa/17,20-liasa (CYP17). Se expresa en las zonas fasciculada y reticular del córtex adrenal y en las gónadas. CYP17 cataliza dos reacciones en la vía esteroidogénica (la 17α-hidroxilación y la 17,20-desmolasa), y cyb5 actúa facilitando la actividad de la reacción 17,20-liasa [16]. Los niños 46,XY presentan genitales ambiguos, con defecto de virilización y metahemoglobinemia [18]. El perfil bioquímico se corresponde con un déficit aislado de 17,20-liasa, con disminución de T y sus precursores (DHEA y androstendiona), aumento de 17OH-P y 17OH-Preg. En la prueba de estimulación con HCG, no aumenta la T y se observa un incremento del cociente entre los esteroides C21 (desoxicortisol) respecto a los esteroides C19 (DHEA y androstendiona). Las gonadotropinas están aumentadas. El diagnóstico molecular muestra la presencia de mutaciones bialélicas en el gen *CYB5A* (Tabla 2).

##### a-9) Déficit en la esteroidogénesis de la vía trasera

Los enzimas de la familia de la aldoreductasa (AKR1C2 y AKR1C4) están implicados en la síntesis de DHT a partir de la P y la 17OH-P por la “via de la puerta trasera”, sin pasar por la síntesis de T. Se han descrito familias con ambigüedad genital en los 46,XY y un fenotipo similar al asociado a déficits de 5α-reductasa tipo 2 ó de P450C17 con déficit aislado de actividad 17,20-liasa, que presentan mutaciones inactivadoras en los genes *AKR1C2* y *AKR1C4* (Tabla 2), lo que indica que la síntesis fetal de DHT a través de la vía alternativa, sin la intermediación de T, también es necesaria para la virilización completa de los genitales externos [19]. La implicación de defectos moleculares en esta vía de la esteroidogénesis también es cuestionada en la génesis del hipospadias que no presenta otras características clínicas o bioquímicas [20].

##### a-10) Déficit de 17β-hidroxi-esteroide deshidrogenasa (17B-HSD) tipo 3

La enzima 17B-HSD cataliza la 17 beta-oxidación y la reducción de esteroides C18 y C19 en varios tejidos. Se han descrito varias isoenzimas y el tipo 3 se expresa en el testículo donde cataliza la conversión de androstendiona en T, de DHEA en 5-androstenediol y de estrona (E1) en estradiol (E2) [21], [22]. El déficit de este enzima se asocia con genitales ambiguos o completamente femeninos. A nivel bioquímico presentan un gran aumento de la concentración de androstendiona con T normal o baja, lo que da lugar a un cociente androstendiona/T muy elevado, tanto basal como tras estimulación con HCG. La DHEA y la DHT pueden estar aumentadas, así como las concentraciones de E1 y E2. En la pubertad aumentan las gonadotropinas [23]. El diagnóstico molecular muestra la presencia de mutaciones bialélicas en el gen *HSD17B3* (Tabla 2).

##### a-11) Déficit de 5α-reductasa tipo 2

Este enzima cataliza el paso de T a DHT que es el andrógeno más potente (se une al mismo receptor, pero con 10 veces más afinidad) y regula la diferenciación del seno urogenital y el tubérculo genital (próstata, cuerpos cavernosos, uretra masculina y bolsas escrotales) [24]. Los niños 46,XY con déficit de este enzima presentan unos genitales prácticamente femeninos al nacer y se les suele asignar sexo femenino. Sin embargo, en la pubertad, aumenta la producción de T que provoca la virilización de los genitales externos con crecimiento del pene y la aparición de características sexuales secundarias con aumento de la masa muscular, masculinización de la voz y aumento en la líbido, excepto la escasa aparición de vello sexual.

A nivel bioquímico presentan una concentración de T normal con aumento del cociente T/DHT basal o tras estimulación con HCG. No hay consenso sobre los valores de corte para este cociente que, es muy variable en niños normales y depende de la edad (más elevado en el lactante) y del estadio puberal. En la prepubertad se han propuesto puntos de corte T/DHT > 25 pero otros autores han reportado valores inferiores (con una media de 12 en pacientes con mutaciones en el gen *SRD5A2*) [25]. Este cociente puede estar también elevado en pacientes con insensibilidad a los andrógenos (por deficiencia relativa en esta actividad enzimática), siendo causa de falsos positivos [26], [27]. Además, los inmunoensayos actuales para la DHT muestran poca sensibilidad y especificidad, por lo que este cociente ya no debería ser utilizado en el diagnóstico diferencial. La mejor sensibilidad y especificidad la proporciona la determinación de metabolitos urinarios de glucocorticoides y andrógenos por cromatografía tándem gases-espectrometría de masas (GC-MS/MS): tanto en edades prepuberales como en el adulto gonadectomizado, la disminución de los cocientes entre los metabolitos 5α y 5β-reducidos (androsterona/etiocolanolona, en el caso de los andrógenos) es patognomónica para el déficit de 5α-reductasa tipo 2 [24], [28].

A nivel molecular, se observan mutaciones bialélicas en el gen *SRD5A2* (Tabla 2) [29].

##### a-12) Hipospadias y/o criptorquidia aislados

La búsqueda de genes implicados en los DSD con cariotipo 46,XY cuando los genes hasta entonces descritos son normales, ha conseguido describir varios genes cuyas mutaciones parecen asociarse al fenotipo. Entre ellos, los más frecuentemente descritos son (Tabla 2):

– *MAMLD1* (*CXOrf6*) localizado en el cromosoma X, por lo que algunos autores denominan el fenotipo como “hipospadias ligado al X” [30], [31]. La patogenicidad de las mutaciones ha sido también discutida y algunos autores opinan que los fenotipos que no muestran unos marcadores bioquímicos específicos podrían ser consecuencia de la suma de variaciones en varios genes cuyos efectos individuales podrían no tener consecuencias fenotípicas [32], [33]^.^
– *ATF3* codifica un factor de transcripción que activa el promotor del gen del receptor de andrógenos (AR). El efecto de sus mutaciones sería dominante [34].
– *INSL3* codifica la proteína INSL3 (proteína 3 similar a la insulina) que ha sido implicada en la regulación del descenso de los testículos a las bolsas escrotales durante el 3er trimestre del desarrollo fetal. Se han descrito mutaciones monoalélicas en casos familiares de criptorquidia aislada [35].
– *RXFP2* (*LGR8/GREAT/GPR106*) ha sido también asociado a la presencia de criptorquidia aislada [34].

#### b) Anomalías en la acción de los andrógenos

El síndrome de insensibilidad a los andrógenos es un trastorno recesivo ligado al cromosoma X que afecta al receptor de andrógenos. Es la causa más frecuente de DSD 46,XY con fenotipo femenino [36]. En las formas completas (CAIS, del inglés *Complete Androgen Insensitivity Syndrome*) el fenotipo presenta genitales externos completamente femeninos, genitales internos masculinos excepto una vagina corta (algunos casos pueden presentar útero) y gónadas que son testículos de estructura normal, pero de localización intraabdominal, que pueden haber sido clasificados como hernias inguinales bilaterales. La actividad de las células de Sertoli y de las de Leydig está preservada por lo que las concentraciones de T y de AMH son normales o elevadas [37]. En la prepubertad la T basal es baja, como corresponde a esta etapa de maduración, pero la respuesta de T al estímulo con HCG es superponible al observado en niños sanos, hallándose incluso en los límites superiores [36]. En la pubertad aumenta la LH, con FSH normal o algo aumentada, y las concentraciones de E2 y de la proteína transportadora de las hormonas sexuales (SHBG) son elevadas para el sexo masculino.

Las formas parciales (PAIS: *Partial Androgen Insensitivity Syndrome*) presentan diferentes grados de ambigüedad genital (hipospadias, micropene, escroto bífido) y un patrón bioquímico menos marcado. Suele haber un aumento de LH y de T durante los primeros meses de vida (minipubertad) como corresponde a un varón normal, patrón que no parece ocurrir en las formas completas (CAIS) [38].

El diagnóstico de CAIS o de PAIS debe ser confirmado molecularmente, detectándose una mutación en el gen *AR* (Tabla 2). La asociación entre el fenotipo clínico y bioquímico y el diagnóstico molecular es muy estrecha en las CAIS, no así en la/os PAIS en los que el porcentaje de diagnósticos moleculares es muy bajo [39]. En algunos casos, se ha podido demostrar que la mutación en el gen *AR* no es germinal sino adquirida, postcigótica, presentándose un mosaicismo celular [40].

Hay pacientes con fenotipo PAIS que algunos autores denominan “idiopáticos” ya que no se ha detectado ninguna alteración genética. En algunos casos se ha demostrado que la causa podía ser la exposición fetal a agentes terapéuticos administrados a la madre (entre ellos el dietilsilbestrol o el clomifeno) o a contaminantes medioambientales que ejercen acciones anti-androgénicas [41], [42], [43], [44].

#### c) Desarrollo genital anómalo por secreción o acción anómala de la AMH

La persistencia aislada de los conductos de Müller puede ser debida a un déficit aislado de AMH por mutaciones inactivadoras en el gen *AMH* o por falta de respuesta a la AMH por mutaciones inactivadoras en el gen que codifica su receptor (*AMHR2*) [45] (Tabla 2). El cuadro clínico de ambos es muy similar. La diferencia entre ambos síndromes es que en las mutaciones del gen *AMH* las concentraciones séricas de AMH son indetectables, mientras que en las mutaciones del receptor son normales o elevadas. La persistencia de los conductos de Müller también se asocia a las disgenesias gonadales (con afectación de las células de Leydig y de Sertoli), por lo que el diagnóstico diferencial debe comportar el análisis de los datos anatómicos, bioquímicos y moleculares.

#### d) Síndromes malformativos complejos

Entre la lista de genes cuyas mutaciones se asocian a un DSD en los 46,XY por provocar grados variables de disgenesia gonadal, algunos se asocian a malformaciones en otros tejidos y sistemas y dan lugar a diversos síndromes (*SOX9, WNT, WT1,* etc.*)* (Tabla 2). Existen, además, muchas otras alteraciones genéticas, tanto estructurales como monogénicas, que se asocian a síndromes complejos que pueden acompañarse de anomalías renales, cardíacas, retraso del crecimiento y mental e incluyen afectación del desarrollo gonadal y/o genital [46]. Entre estos síndromes podemos mencionar los síndromes de Aarskog, de Robinow, las anomalías del desarrollo cloacal (Tabla 1).

Por último, cabe mencionar que en los fetos 46,XY con retraso intrauterino del crecimiento de inicio precoz y severo (Tabla 1), que presentan hipospadias con mayor frecuencia que los niños con peso adecuado y en los que se han descartado anomalías genéticas, se ha propuesto que la causa puede ser una anomalía en la cronología del desarrollo sexual [47].

## IV. Propuestas para el diagnóstico diferencial de los DSD (papel de la bioquímica y de la genética)

El diagnóstico diferencial de la etiología de un DSD debe ser multidisciplinar involucrando varias especialidades médicas entre las que se hallan la bioquímica y la genética. El clínico responsable de la coordinación del equipo depende de la edad de la persona a estudiar y del motivo de consulta. La bioquímica debe analizar parámetros generales, hormonas proteicas en sangre y esteroides en sangre y/o en orina; las condiciones, basales o en pruebas dinámicas de estimulación, y las hormonas a analizar dependerán de la edad (RN, lactante, niño prepúber, adolescente o adulto), de la clínica acompañante y del cariotipo. La exploración genética debe siempre comenzar por un cariotipo, al que podrán seguir análisis moleculares en caso de que se oriente el diagnóstico hacia una causa monogénica. Se han publicado numerosos algoritmos diagnósticos, dependiendo del cariotipo, de la presencia o no de gónadas palpables, de la ecografía abdominal y de las hormonas medidas [48], [49], [50], [51], [52].

La orientación del diagnóstico molecular debe ser paralela a las informaciones proporcionadas por las diversas técnicas aplicadas. Las técnicas actuales de secuenciación masiva (paneles de genes candidatos o exoma completo) permiten una primera aproximación más rápida hacia las causas más frecuentes y la revisión posterior de los resultados para analizar otras causas menos frecuentes o nuevas [53], [54], [55], [56], [57].

**Figura 1:**
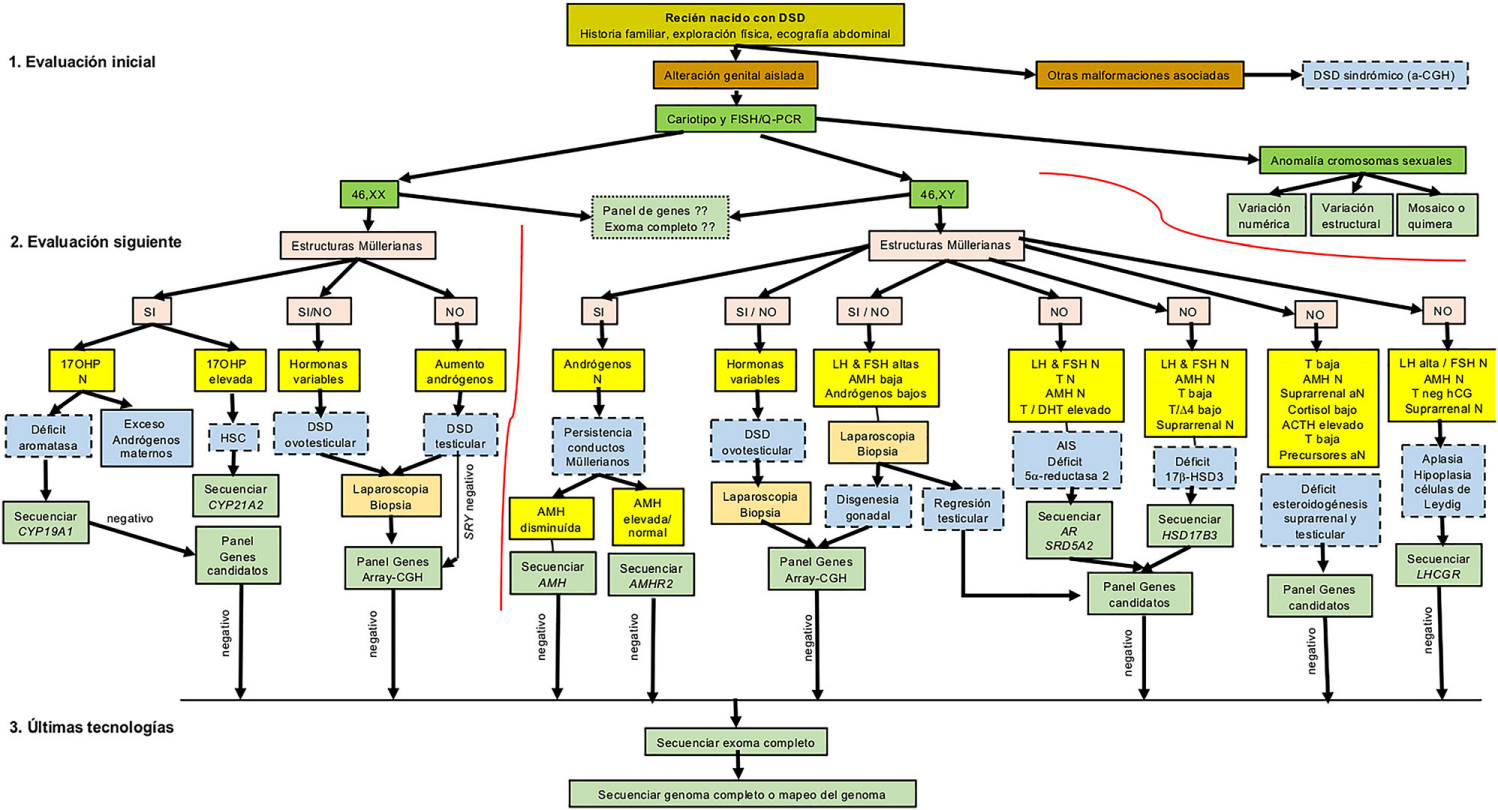
Algoritmo diagnóstico en un recién nacido que presente un desarrollo sexual anómalo o diferente (DSD). Si existen otras malformaciones, se trata de un DSD sindrómico y se realizará un análisis de hibridación comparativa del genoma (a-CGH). Cuando la anomalía genital es aislada, el cariotipo subclasifica el DSD en tres grupos diagnósticos: 46,XX, 46,XY o anomalías de los cromosomas sexuales. La evaluación en los grupos 46,XX y 46,XY depende de la presencia o no de estructuras Müllerianas (ecografía abdominal) y los resultados hormonales que permiten orientar los distintos grupos diagnósticos y los genes candidatos a analizar. Éstos pueden ser analizados individualmente o mediante secuenciación masiva de paneles de genes candidatos o mediante la secuenciación de un exoma o de un genoma completo. DSD, desarrollo sexual anómalo o diferente; 17OH-P, 17-hidroxi-progesterona; N, normal; LH, hormona luteinizante; FSH, hormona folículo estimulante; AMH, hormona anti-Mülleriana; T, testosterona; DHT, dihidrotestosterona; Δ4, androstendiona; ACTH, hormona adrenocorticotropa; T neg HCG, falta de respuesta de T al test de hCG; HSC, hiperplasia suprarrenal congénita; AIS, síndrome de insensibilidad a los andrógenos.

La evaluación inicial incluirá la historia familiar, la exploración física y una ecografía abdominal (Figura 1).

Si, además de la malformación genital, existen otras malformaciones, se trata de un DSD sindrómico y se realizará un análisis de hibridación comparativa del genoma (a-CGH) al que seguirán otros estudios genéticos.

Cuando la anomalía genital es aislada, el cariotipo subclasifica el DSD en uno de los tres grupos diagnósticos: 46,XX, 46,XY o anomalías de los cromosomas sexuales. Éste último grupo se subclasifica según si la variación es numérica, estructural o si existen mosaicos.

Los datos aportados por la ecografía abdominal (presencia de estructuras Müllerianas), los resultados hormonales y la laparoscopia con la biopsia, orientarán hacia los genes candidatos a analizar individualmente, mediante secuenciación masiva de paneles de genes candidatos, del exoma completo o del genoma completo.
